# Explainable machine learning model to predict refeeding syndrome in patients with severe acute pancreatitis

**DOI:** 10.3389/fnut.2025.1741052

**Published:** 2026-01-23

**Authors:** Cui Wu, Shuangshuang Jing, Dinghui Guo, Congcong Cheng, Sujuan Fei

**Affiliations:** 1 Department of Nursing, Xuzhou Tongshan District Hospital of Traditional Chinese Medicine, Xuzhou, Jiangsu, China; 2 Xuzhou Medical University, Xuzhou, Jiangsu, China; 3 Department of Gastroenterology, The Affiliated Hospital of Xuzhou Medical University, Xuzhou, Jiangsu, China

**Keywords:** gradient boosting machine, machine learning, prediction model, refeeding syndrome, severe acute pancreatitis, SHAP analysis

## Abstract

**Objective:**

To construct and validate a risk prediction model for refeeding syndrome (RFS) in patients with severe acute pancreatitis (SAP), identify high-risk individuals before overt electrolyte abnormalities occur, and provide decision support for the timing of enteral nutrition initiation and early personalized intervention.

**Methods:**

A retrospective cohort study was conducted on SAP patients admitted to Xuzhou Medical University Affiliated Hospital (XYFY) between September 2018 and September 2025. Patients were divided into RFS and Non-RFS groups based on the development of RFS after initiating enteral nutrition. Clinical data differences between groups were compared, least absolute shrinkage and selection operator (LASSO) regression was used for feature selection, and six machine learning (ML) algorithms were applied to build prediction models. Model performance was evaluated using receiver operating characteristic (ROC) curves, calibration curves, and decision curves. SHapley Additive exPlanations (SHAP) analysis was performed to interpret the contribution of key features.

**Results:**

Seven predictive features were identified for model construction. The gradient boosting machine (GBM) model exhibited good generalization ability, with area under the curve (AUC) values of 0.851 (95% CI: 0.809–0.894) in the training set and 0.762 (95% CI: 0.672–0.852) in the testing set. Calibration curves confirmed consistency between predicted probabilities and actual outcomes, while decision curves demonstrated favorable net benefits across different clinical decision thresholds. SHAP analysis ranked feature importance as follows: serum potassium (K), serum sodium (Na), serum calcium (Ca), gastrointestinal decompression, blood urea nitrogen (BUN), diabetes mellitus (DM) history, and diuretic use.

**Conclusion:**

The GBM model effectively predicts RFS risk in SAP patients after initiating enteral nutrition.

## 1 Introduction

Refeeding syndrome (RFS) is a severe metabolic complication characterized by profound electrolyte disturbances, fluid imbalance, and organ dysfunction. It occurs when nutritional support is restarted in patients with prolonged fasting or severe malnutrition, driven by core pathophysiological changes that result from rapid insulin secretion recovery and subsequent intracellular electrolyte shifts (1). In critically ill patients, RFS is closely associated with increased mortality and prolonged hospital stays (2). Although consensus definitions and high-risk population criteria have been proposed by institutions such as the American Society for Parenteral and Enteral Nutrition (ASPEN) (3, 4), early identification remains challenging in clinically complex populations.

Patients with severe acute pancreatitis (SAP) are at extremely high risk of RFS. On one hand, SAP-induced systemic inflammatory response, hypermetabolic consumption, and gastrointestinal dysfunction rapidly lead to malnutrition (5, 6). On the other hand, early enteral nutrition—the cornerstone of SAP treatment—serves as a classic trigger for RFS (7). However, existing RFS prediction models primarily focus on general intensive care unit (ICU) or hospital-wide populations (8–10). Their predictive variables (e.g., specific urinary biomarkers) or targets (e.g., isolated hypophosphatemia) lack applicability and specificity in the unique pathophysiological context of SAP. Thus, developing an interpretable RFS risk prediction tool tailored to SAP patients, based on routine clinical data, is crucial for precise early warning and personalized nutrition management.

Recently, machine learning (ML) has demonstrated significant advantages in medical prediction models. It captures complex non-linear relationships between variables, potentially outperforming traditional logistic regression (LR) (11, 12). Building on this, we systematically collected clinical data from SAP patients, constructed RFS risk prediction models using multiple ML algorithms, and enhanced model interpretability via SHapley Additive exPlanations (SHAP) analysis. The present study aims to develop a dedicated RFS risk early warning tool for SAP patients, with a core focus on identifying high-risk individuals through routine clinical indicators before the initiation of nutritional support. It targets the state where RFS risk exists without overt electrolyte abnormalities, rather than diagnosing existing electrolyte disturbances or confirmed RFS, ultimately providing data support for the clinical development of individualized monitoring and preventive intervention plans.

## 2 Materials and methods

### 2.1 Data resource

This single-center retrospective cohort study included SAP patients admitted to Xuzhou Medical University Affiliated Hospital (XYFY) from September 2018 to September 2025. Patients were categorized into RFS and Non-RFS groups based on RFS development after initiating enteral nutrition. Electronic medical record data were collected for model construction and validation. The study adhered to the Declaration of Helsinki and was approved by the Ethics Committee of Xuzhou Medical University Affiliated Hospital (Ethics No.: XYFY2025-KL236-01). Written informed consent was waived due to the retrospective nature and de-identified patient data.

### 2.2 Inclusion and exclusion criteria

**Inclusion criteria:** (1) SAP patients who received enteral nutrition during hospitalization. In addition to meeting acute pancreatitis (AP) diagnostic criteria (13), patients must satisfy one of the following: Meeting revised Atlanta classification (RAC) severe criteria (14), i.e., organ dysfunction lasting >48 h. Organ dysfunction was defined using the modified Marshall scoring system (15), with any organ score ≥2. Specifically, organ dysfunction was present if oxygenation index < 300, serum creatinine (Scr) > 170 μmol/L, or systolic blood pressure < 90 mmHg unresponsive to fluid resuscitation; Inability to obtain the complete above data, but ICU stay > 48 h.

**Exclusion criteria:** Age < 18 years; Hospital stay < 48 h; Concurrent pregnancy or malignant tumors; Risk factors for hypophosphatemia (e.g., hemodialysis, treatment for hyperphosphatemia); History of parathyroidectomy; Missing serum electrolyte data; Mild and moderately severe acute pancreatitis. This study focuses on SAP patients because they exhibit more prominent systemic inflammatory response, hypermetabolic consumption, and gastrointestinal dysfunction, making them an extremely high-risk population for RFS. Currently, there is a lack of targeted prediction tools for this group. Excluding mild and moderately severe cases aims to ensure the homogeneity of the study cohort, minimize confounding effects introduced by differences in disease severity, and thus enable the constructed model to more accurately reflect the unique RFS risk characteristics of the high-risk SAP population.

### 2.3 Research variables

(1) Demographic data: Age, gender, body mass index (BMI), smoking history, drinking history, Nutritional Risk Screening (NRS) score, pancreatitis etiology classification;
(2) Comorbidities: Hypertension (HTN), diabetes mellitus (DM), liver disease (LD), chronic kidney disease (CKD);
(3) Pre-enteral nutrition treatments: Mechanical ventilation (MV), renal replacement therapy (RRT), gastrointestinal decompression, medication use (insulin, diuretics, glucocorticoids, etc.);
(4) Laboratory results before initiating enteral nutrition (last measurement): Total bilirubin (TBIL), alkaline phosphatase (ALP), prealbumin (PALB), albumin (ALB), blood urea nitrogen (BUN), serum creatinine (Scr), total cholesterol (Chol), triglycerides (TG), serum potassium (K), serum sodium (Na), serum magnesium (Mg), serum calcium (Ca), serum phosphorus (P).

### 2.4 Variable definitions

(1) Refeeding syndrome (RFS): Defined per 2020 ASPEN criteria (3). RFS was diagnosed if one or more serum electrolytes (phosphorus, potassium, or magnesium) decreased within 5 days of increasing energy intake, with any two electrolytes declining by ≥10% from pre-refeeding levels to ensure accuracy. Compared with the 2020 ASPEN criteria, this study adds a strict requirement of at least a 10% decrease in two or more electrolytes. The purpose is to improve the specificity of RFS diagnosis and avoid misclassifying non-nutrition-related electrolyte fluctuations as RFS. It should be noted that the definition in this study differs from some previous research. This is mainly because SAP patients have unique pathophysiological characteristics, and conventional criteria are prone to interference from non-nutritional factors. While the strict definition may underestimate mild or early cases, it better aligns with the actual clinical needs of SAP management.
(2) Nutritional Risk Screening (NRS) (16): This indicator is assessed upon patient admission. The tool includes three dimensions: age, nutritional status, and physical condition. Age ≥ 70 years = 1 point (< 70 = 0); nutritional status: normal = 0, 5% weight loss in 3 months or 50–75% normal food intake in 1 week = 1, 5% weight loss in 2 months or BMI 18.5–20.5 or 25–50% normal food intake in 1 week = 2, 5% weight loss in 1 month or BMI < 18.5 or < 25% normal food intake in 1 week = 3; physical condition: normal = 0, ambulatory but weak = 1, bedridden = 2, critically ill = 3. Total scores range from 0 to 7, with higher scores indicating greater nutritional risk.
(3) Pancreatitis etiology classification: (a) Biliary: Gallbladder/biliary stones on imaging plus abnormal liver function; (b) Alcoholic: Heavy drinking or long-term alcohol use (≥50 ml/d for ≥ 5 years) after excluding other causes; (c) Hypertriglyceridemic: Serum triglycerides >5.65 mmol/L; (d) Other: Not meeting the above three etiologies.

### 2.5 Model construction and evaluation

Missing data were handled using multiple imputation (17). Patients were randomly divided into training (70%) and testing (30%) sets for model training and validation. Least absolute shrinkage and selection operator (LASSO) regression was used for feature selection, with 10-fold cross-validation to evaluate performance and obtain a streamlined feature set.

Six ML algorithms were employed: logistic regression (LR), support vector machine (SVM), random forest (RF), gradient boosting machine (GBM), extreme gradient boosting (XGBoost), and elastic network (EN). Nested 10-fold cross-validation was used for hyperparameter optimization (HPO) to enhance model reliability.

Model performance was evaluated via: Discrimination: Area under the curve (AUC) of receiver operating characteristic (ROC) curves and confusion matrix-derived metrics (sensitivity, specificity, accuracy, precision, Youden index, F1 score); Calibration: Calibration curves and Hosmer–Lemeshow (H–L) test; Clinical applicability: Decision curves.

### 2.6 Model interpretation and visualization

SHAP analysis was used to quantify and interpret feature importance in the optimal model (18). Specifically: Bar plots for global feature importance ranking; Beeswarm plots for SHAP value distribution across all samples; Partial dependence plots for non-linear relationships between single features and model outputs; Waterfall plots for decomposing individual sample prediction processes.

### 2.7 Statistical analysis

**Figure 1:**
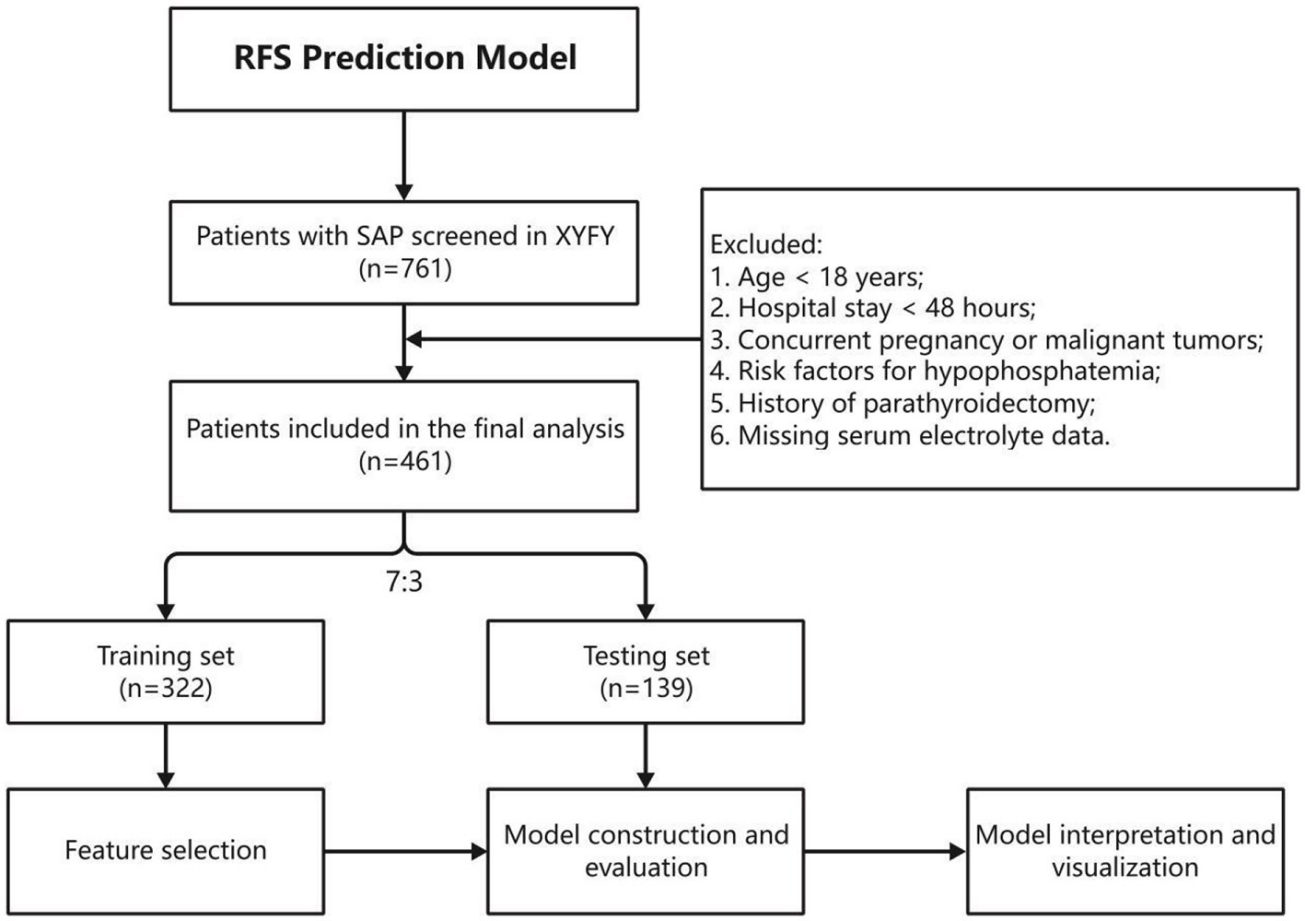
Flowchart of the study. RFS, refeeding syndrome; SAP, severe acute pancreatitis; XYFY, the Affiliated Hospital of Xuzhou Medical University.

All statistical analyses, model construction, and visualization were performed using *R 4.4.1*. Normally distributed continuous variables are presented as mean ± standard deviation (Mean ± SD) with *t*-tests for group comparisons; non-normally distributed variables as median (interquartile range) [Median (IQR)] with Mann–Whitney U-tests. Categorical variables are presented as *n* (%) with chi-square or Fisher's exact tests. A two-tailed *P* < 0.05 was considered statistically significant. The study design is shown in Figure 1.

## 3 Results

### 3.1 Baseline characteristics of the study participants

A total of 461 SAP patients were included, with 166 (36.01%) developing RFS. Compared with the Non-RFS group, RFS patients had higher proportions of DM and CKD, more frequent use of gastrointestinal decompression and diuretics before enteral nutrition, lower TBIL levels, and higher BUN, serum potassium, and serum sodium levels (*P* < 0.05; Table 1). Patients were randomly divided into training (*n* = 322) and testing (*n* = 139) sets, with balanced baseline characteristics between groups (*P* > 0.05; Supplementary Table S1).

**Table 1:** Baseline characteristics of SAP patients stratified by RFS.

**Variables**	**Total (*n* = 461)**	**Non-RFS group (*n* = 295)**	**RFS group (*n* = 166)**	***P-*value**
**Demographic**				
Age, median [IQR]	42.00 [33.00, 56.00]	42.00 [33.00, 55.00]	42.00 [34.00, 60.75]	0.462
Gender, female, *n* (%)	172 (37.31)	107 (36.27)	65 (39.16)	0.539
BMI, median [IQR]	26.78 [24.22, 29.80]	26.84 [24.44, 29.78]	26.57 [24.22, 29.95]	0.402
Smoking history, *n* (%)	102 (22.13)	68 (23.05)	34 (20.48)	0.524
Alcohol consumption, *n* (%)	108 (23.43)	75 (25.42)	33 (19.88)	0.177
NRS, median [IQR]	0.00 [0.00, 0.00]	0.00 [0.00, 0.00]	0.00 [0.00, 0.00]	0.115
**Comorbidities**, ***n*** **(%)**				
Hypertension	151 (32.75)	91 (30.85)	60 (36.14)	0.245
Diabetes	163 (35.36)	87 (29.49)	76 (45.78)	**< 0.001**
LD	8 (1.74)	7 (2.37)	1 (0.60)	0.205
CKD	23 (4.99)	8 (2.71)	15 (9.04)	**0.003**
**Treatments**, ***n*** **(%)**				
MV	59 (12.80)	32 (10.85)	27 (16.27)	0.095
RRT	172 (37.31)	104 (35.25)	68 (40.96)	0.224
Decompression	132 (28.63)	66 (22.37)	66 (39.76)	**< 0.001**
Insulin	285 (61.82)	176 (59.66)	109 (65.66)	0.203
Diuretics	119 (25.81)	61 (20.68)	58 (34.94)	**< 0.001**
Glucocorticoids	86 (18.66)	52 (17.63)	34 (20.48)	0.450
**Type**, ***n*** **(%)**				
Biliary	210 (45.55)	143 (48.47)	67 (40.36)	0.286
Alcoholic	23 (4.99)	13 (4.41)	10 (6.02)	
Hyperlipidemic	90 (19.52)	58 (19.66)	32 (19.28)	
Other	138 (29.93)	81 (27.46)	57 (34.34)	
**Laboratory results, median [IQR]**				
TBIL, μmol/L	14.00 [8.40, 23.40]	15.30 [9.40, 23.80]	11.80 [6.43, 21.95]	**0.005**
ALP, U/L	82.00 [62.00, 108.00]	81.00 [62.00, 104.00]	84.50 [63.00, 119.00]	0.144
PALB, g/L	0.11 [0.08, 0.14]	0.11 [0.08, 0.14]	0.11 [0.07, 0.16]	0.788
ALB, g/L	32.70 [30.10, 35.40]	32.80 [30.40, 35.50]	32.40 [29.92, 35.00]	0.254
BUN, mmol/L	5.20 [3.49, 8.26]	4.77 [3.30, 6.65]	6.47 [3.98, 10.98]	**< 0.001**
Scr, μmol/L	53.00 [41.00, 69.00]	52.00 [42.00, 67.00]	56.00 [41.00, 85.00]	0.096
Chol, mmol/L	4.56 [3.38, 6.38]	4.56 [3.49, 6.39]	4.53 [3.03, 6.19]	0.308
TG, mmol/L	3.11 [1.56, 4.93]	3.15 [1.56, 5.32]	3.08 [1.57, 4.48]	0.414
K, mmol/L	3.79 [3.45, 4.13]	3.65 [3.40, 3.99]	3.99 [3.57, 4.36]	**< 0.001**
Na, mmol/L	138.10 [135.30, 142.50]	137.40 [135.00, 140.60]	140.65 [136.12, 145.67]	**< 0.001**
Mg, mmol/L	0.83 [0.74, 0.92]	0.83 [0.74, 0.91]	0.85 [0.74, 0.95]	0.128
Ca, mmol/L	2.01 [1.73, 2.13]	2.03 [1.81, 2.14]	1.98 [1.43, 2.12]	0.164
P, mmol/L	0.80 [0.54, 1.02]	0.79 [0.54, 1.00]	0.81 [0.54, 1.04]	0.255

SAP, severe acute pancreatitis; RFS, refeeding syndrome; IQR, interquartile range; BMI, body mass index; NRS, nutritional risk screening; LD, liver disease; CKD, chronic kidney disease; MV, mechanical ventilation; RRT, renal replacement therapy; TBIL, total bilirubin; ALP, alkaline phosphatase; PALB, prealbumin; ALB, albumin; BUN, blood urea nitrogen; Scr, serum creatinine; Chol, total cholesterol; TG, triglycerides; K, potassium; Na, sodium; Mg, magnesium; Ca, calcium; P, phosphate. The bold *P*-values are considered to represent statistically significant differences.

### 3.2 Model construction and validation

**Figure 2:**
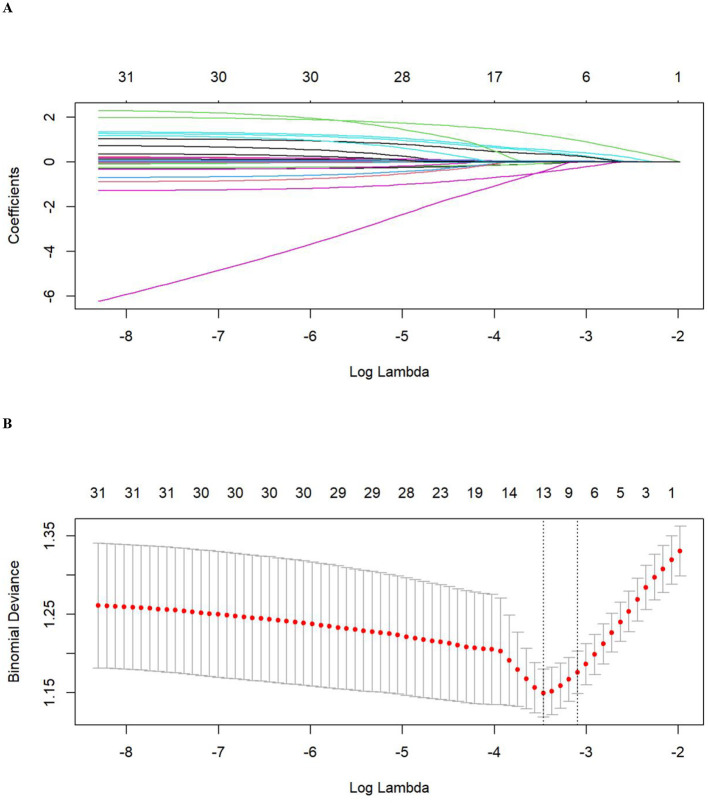
Process of feature variable selection. **(A)** LASSO regression coefficient path plot; **(B)** LASSO binomial deviance plot.

**Figure 3:**
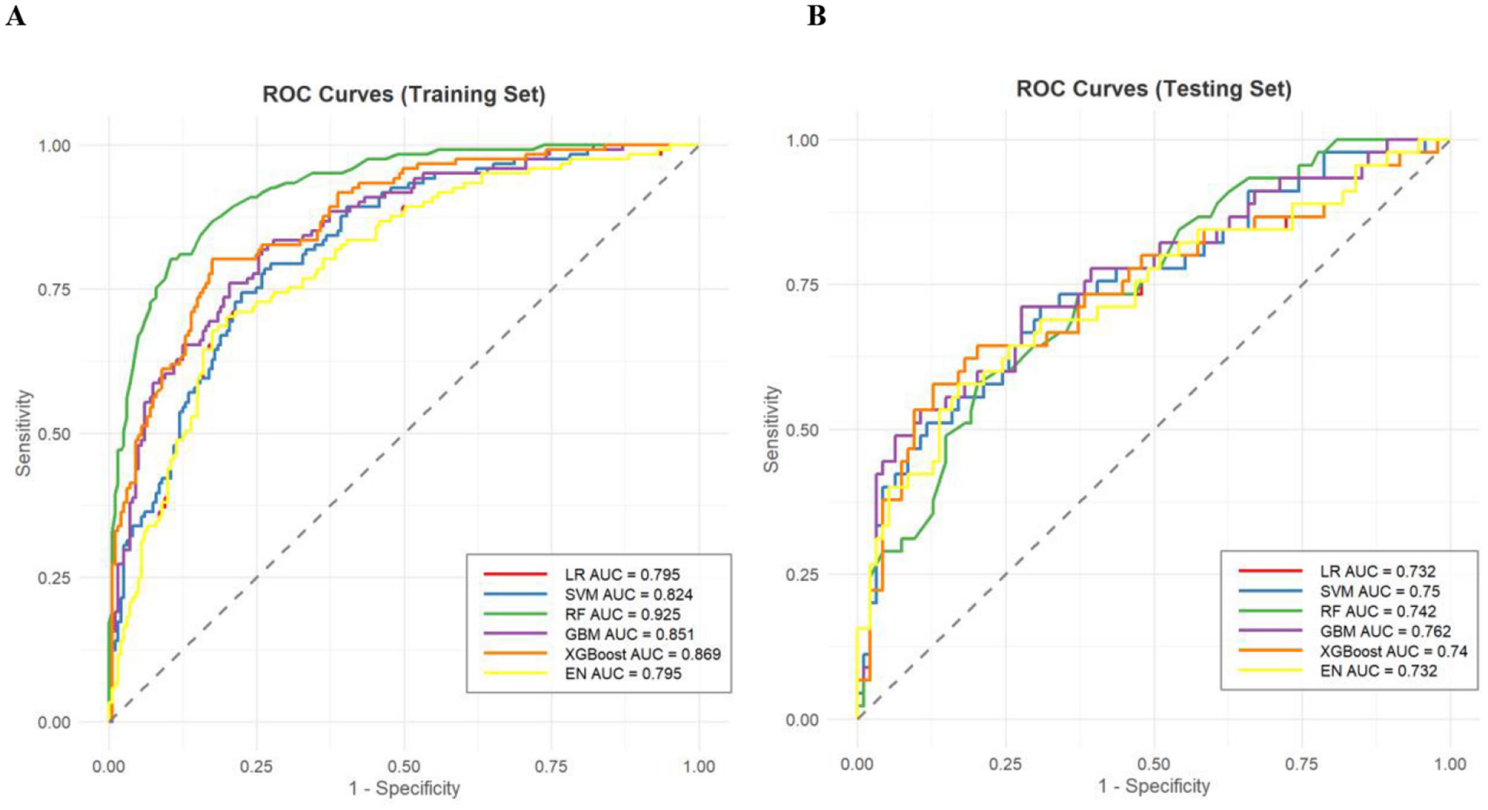
**(A)** ROC curves of each model in the training set. **(B)** ROC curves of each model in the testing set. ROC, receiver operating characteristic; AUC, area under the curve; LR, logistic regression; SVM, support vector machine; RF, random forest; GBM, gradient boosting machine; XGBoost, eXtreme gradient boosting; EN, elastic network.

LASSO regression identified seven optimal predictors: serum potassium, serum sodium, serum calcium, gastrointestinal decompression, BUN, DM history, and diuretic use (Figure 2). Results of ROC curves (Figure 3) showed that the RF model performed the best in the training set with an AUC of 0.925 (95% CI: 0.897–0.954). However, its AUC decreased significantly to 0.742 in the testing set, suggesting a potential overfitting tendency that impairs its extrapolation performance. In contrast, the GBM model exhibited robust and balanced predictive performance in both the training and testing sets, with AUC values of 0.851 and 0.762, respectively. Its performance on the testing set was the best among all models, demonstrating superior generalization ability. Comprehensive evaluation metrics for all models are presented in Table 2.

**Table 2:** Comparison of the performance of each model in the training set and testing set.

**Dataset**	**ML**	**AUC (95%CI)**	**Sensitivity**	**Specificity**	**Accuracy**	**Precision**	**Youden index**	**F1 score**
Training set	LR	0.795 (0.745–0.846)	0.678	0.826	0.770	0.701	0.504	0.689
	SVM	0.824 (0.779–0.870)	0.785	0.736	0.755	0.642	0.521	0.706
	RF	0.925 (0.897–0.954)	0.802	0.896	0.860	0.822	0.697	0.812
	GBM	0.851 (0.809–0.894)	0.818	0.741	0.770	0.656	0.559	0.728
	XGBoost	0.869 (0.830–0.909)	0.802	0.826	0.817	0.735	0.628	0.767
	EN	0.795 (0.745–0.846)	0.678	0.826	0.770	0.701	0.504	0.689
Testing set	LR	0.732 (0.636–0.827)	0.578	0.830	0.748	0.619	0.408	0.598
	SVM	0.750 (0.659–0.840)	0.711	0.691	0.698	0.525	0.403	0.604
	RF	0.742 (0.656–0.829)	0.578	0.798	0.727	0.578	0.376	0.578
	GBM	0.762 (0.672–0.852)	0.711	0.723	0.719	0.552	0.435	0.622
	XGBoost	0.740 (0.644–0.836)	0.578	0.872	0.777	0.684	0.450	0.627
	EN	0.732 (0.636–0.828)	0.578	0.830	0.748	0.619	0.408	0.598

ML, machine learning; AUC, area under the curve; CI, confidence interval; LR, logistic regression; SVM, support vector machine; RF, random forest; GBM, gradient boosting machine; XGBoost, eXtreme gradient boosting; EN, elastic network.

### 3.3 Model calibration and clinical applicability

**Figure 4:**
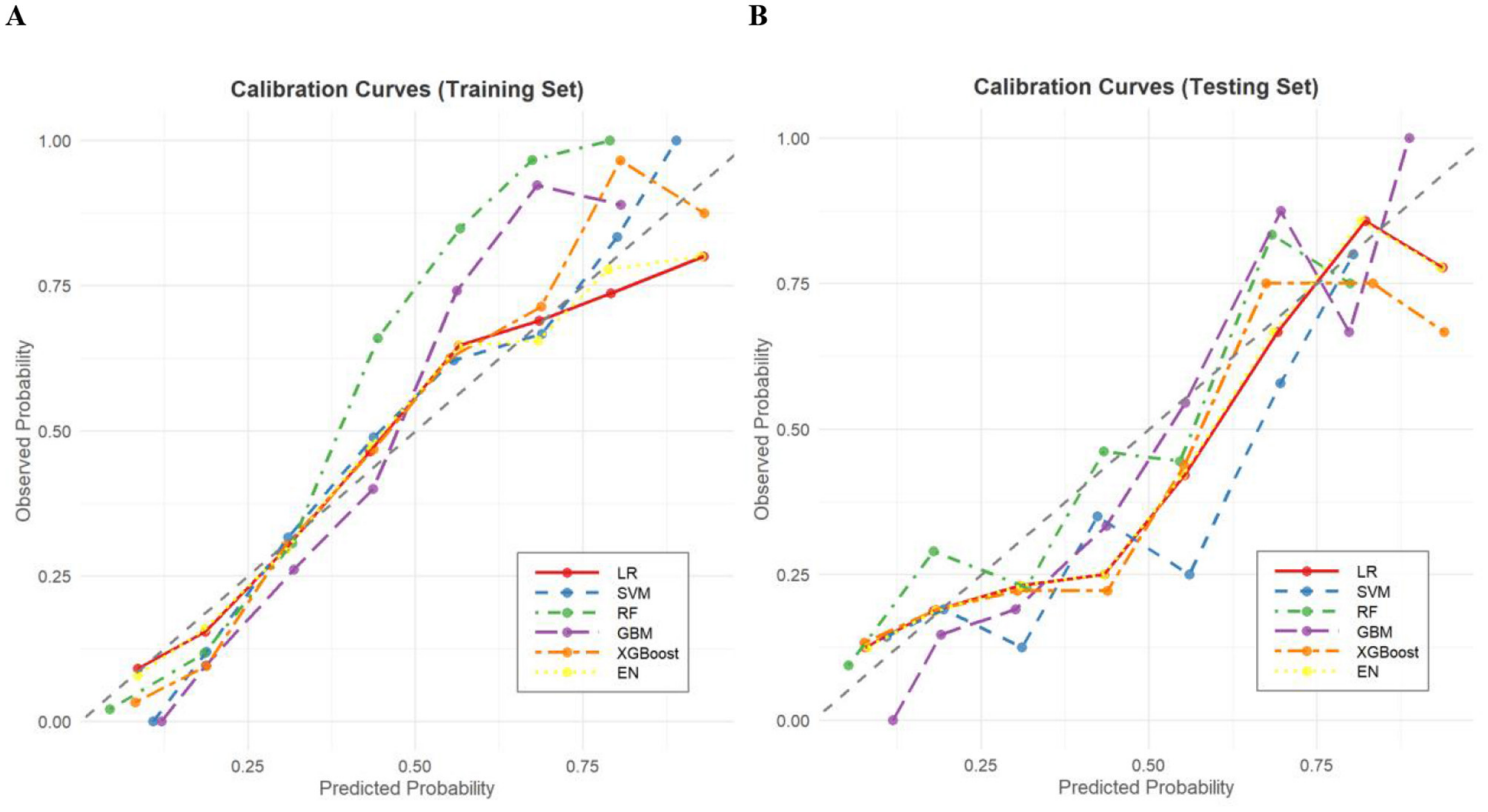
**(A)** Calibration curves of each model in the training set. **(B)** Calibration curves of each model in the testing set. LR, logistic regression; SVM, support vector machine; RF, random forest; GBM, gradient boosting machine; XGBoost, eXtreme gradient boosting; EN, elastic network.

**Figure 5:**
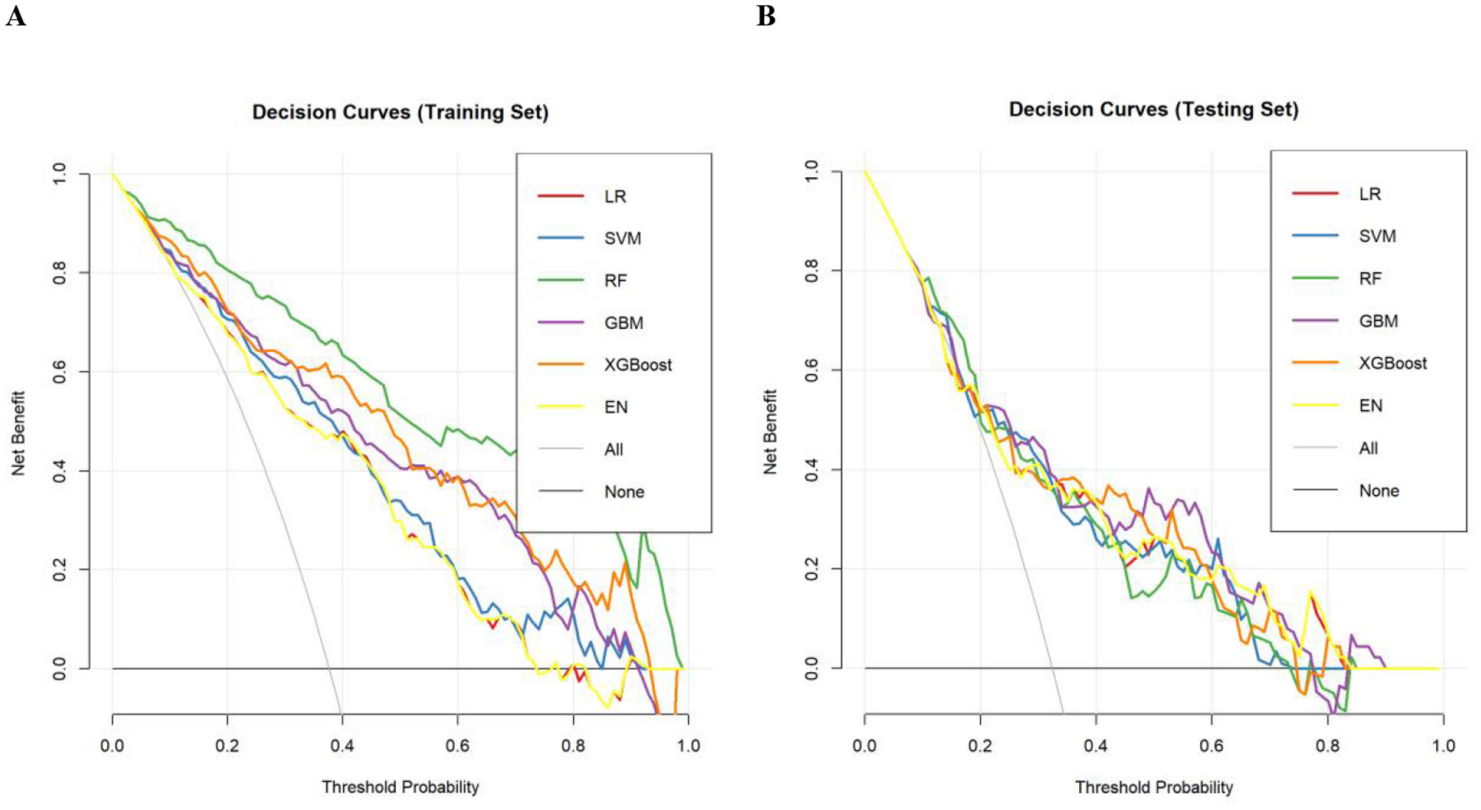
**(A)** Decision curves of each model in the training set. **(B)** Decision curves of each model in the testing set. The net benefit of each model at different threshold probabilities for predicting RFS in SAP patients. LR, logistic regression; SVM, support vector machine; RF, random forest; GBM, gradient boosting machine; XGBoost, eXtreme gradient boosting; EN, elastic network.

Calibration curves for both training and testing sets showed good agreement between predicted and actual probabilities (Figure 4). H–L tests in the testing set yielded *P* > 0.05 for all models, indicating no significant calibration bias (Supplementary Table S2). Decision curves demonstrated high net benefits for all models in both training and testing sets, confirming clinical applicability (Figure 5).

After a comprehensive consideration of the overall performance, calibration status, and clinical applicability of each model, the GBM model exhibited the most robust generalization ability in the testing set. Its calibration curves and decision curves demonstrated good calibration and net benefit, so it was identified as the optimal model and used for subsequent SHAP analysis.

### 3.4 Model interpretation and visualization

**Figure 6:**
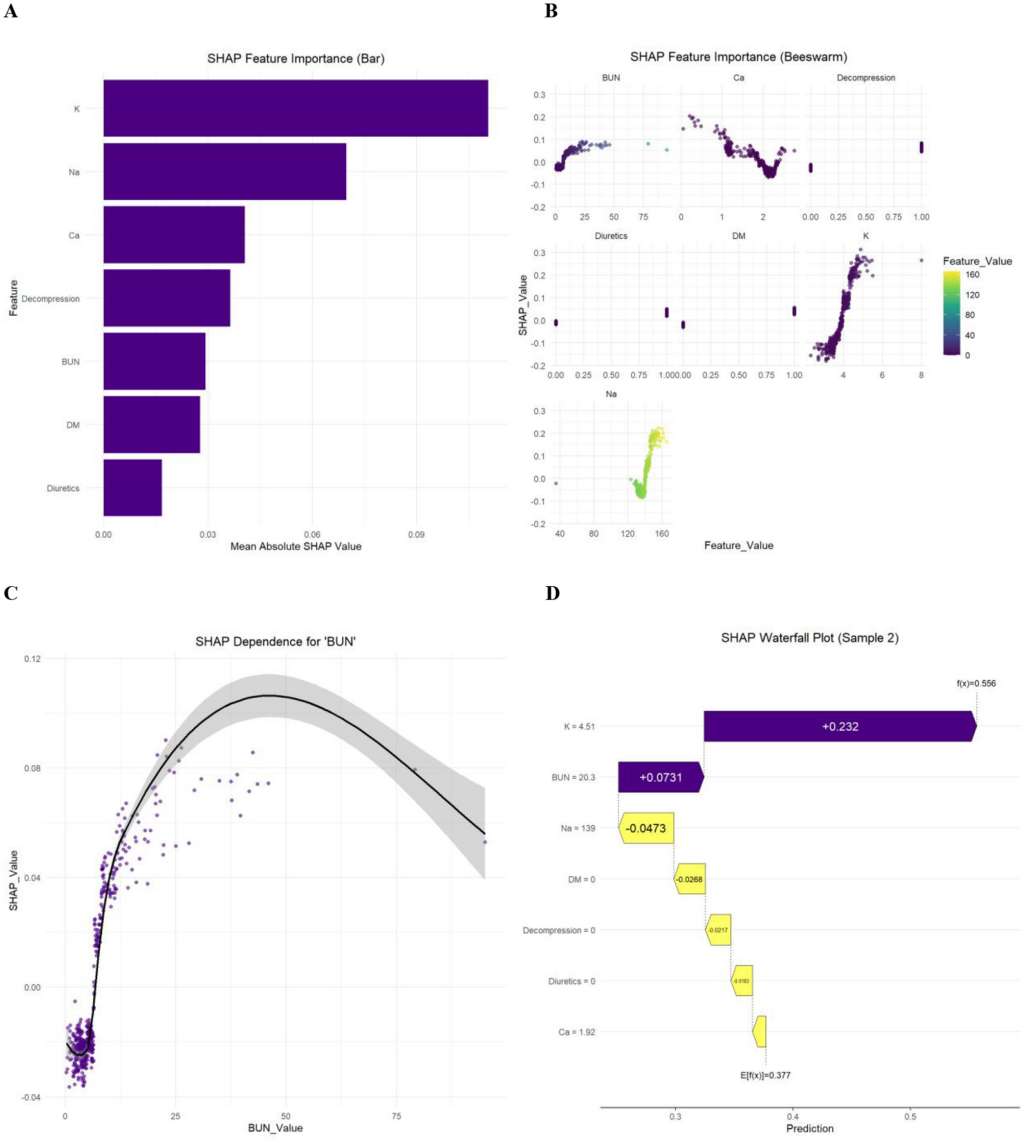
SHAP analysis visualizations for interpreting feature contributions in predicting RFS in SAP patients. **(A)** Bar plot of mean absolute SHAP values, showing the average impact magnitude of each feature on model predictions; **(B)** beeswarm plot of SHAP values, illustrating the distribution of feature impacts across different feature values; **(C)** dependency plot, showing the relationship between BUN and its SHAP value for SAP patients' RFS prediction; **(D)** waterfall plot for a single prediction instance, decomposing the model output into individual feature contributions. SHAP, SHapley additive explanations; K, serum potassium; Na, serum sodium; Ca, serum calcium; BUN, blood urea nitrogen; DM, diabetes mellitus.

SHAP analysis of the GBM model clarified feature importance and contributions: Feature importance ranking: serum potassium > serum sodium > serum calcium > gastrointestinal decompression > BUN > DM history > diuretic use (Figure 6A); Beeswarm plots demonstrates that patients with a history of DM, gastrointestinal decompression and diuretic use before the initiation of enteral nutrition, and low serum calcium as well as high blood urea nitrogen, serum potassium, and serum sodium within the normal range have a significantly increased risk of RFS (Figure 6B); Partial dependence plots illustrated the association between BUN levels and RFS risk prediction (Figure 6C); Waterfall plots visualized the prediction process for individual samples (e.g., Sample 2), showing the specific impact of each feature on RFS risk (Figure 6D).

## 4 Discussion

This study constructed and validated RFS risk prediction models for SAP patients using six ML algorithms based on single-center retrospective data. The GBM model showed good predictive performance and generalization ability, with seven easily accessible clinical features identified via LASSO regression and SHAP analysis. This provides a reliable tool for early RFS identification in SAP patients.

The seven identified predictors have clear clinical and pathophysiological significance. Electrolyte disturbances are central to RFS (19), and our model highlights high pre-refeeding serum potassium/sodium and low serum calcium as key warning signs. In SAP patients, elevated pre-refeeding serum potassium/sodium may reflect electrolyte distribution abnormalities or impaired excretion due to stress, dehydration, or underlying renal dysfunction (20). This “apparently normal or elevated” state often masks true electrolyte depletion, leading clinicians to overlook potential risks without targeted supplementation during enteral nutrition initiation. Once enteral nutrition starts, restored insulin secretion drives rapid intracellular shifts of potassium, phosphorus, and magnesium, causing acute serum depletion and typical RFS manifestations (21). Hypocalcemia is common in SAP, associated with peripancreatic fat necrosis (calcium soap formation), hypomagnesemia, and vitamin D metabolism disorders (22); refeeding further exacerbates calcium-phosphorus imbalance (23), increasing neuromuscular instability. Beyond electrolytes, DM history and diuretic use directly elevate metabolic imbalance risk by impairing insulin sensitivity and promoting renal electrolyte loss. Elevated BUN indicates dehydration or hypercatabolism, a marker of systemic metabolic disturbance (24). Finally, gastrointestinal decompression—an important procedure-related factor—signals severe gastrointestinal dysfunction and exacerbates electrolyte loss via digestive fluid depletion (25), compounding metabolic challenges during refeeding.

It is important to note that the core function of this model lies in risk early warning and stratification, and its prediction logic and clinical application require an in-depth understanding through the following three aspects. First, SHAP analysis (Figure 6B) reveals that key predictors such as serum potassium, serum sodium, serum calcium, and blood urea nitrogen affect RFS risk mainly through their relative fluctuations within the normal range. For SAP patients with laboratory indicators within the normal range, serum potassium or sodium close to the upper limit, or serum calcium tending to the lower limit, may indicate a pseudo-normal state of intracellular electrolyte depletion or potential metabolic disorders. The model can sensitively capture such subtle changes, assisting clinicians to strengthen monitoring or implement preventive interventions before initiating enteral nutrition. Second, diuretic use not only reflects the volume management needs or comorbidities (such as heart failure) of SAP patients but may also cause electrolyte loss through promoting renal excretion, thereby increasing the risk of RFS. Through feature integration in the multivariate machine learning model, this study has controlled its confounding effect at the statistical level. However, due to the retrospective nature of the study, details such as the specific types, dosages, and clear indications of diuretics could not be collected. When applying this model clinically, the contribution of this predictor needs to be comprehensively interpreted in combination with the patient's volume status, comorbidities, and medication background. Additionally, NRS scores assessed at admission were generally low in this study. The core reason is that the score is designed around chronic nutritional depletion indicators, while the nutritional risk of SAP patients stems from acute stress response, gastrointestinal dysfunction, and hypermetabolic state. Such acute risks are difficult to capture by traditional scoring tools relying on chronic indicators. This phenomenon further confirms the limited applicability of traditional nutritional risk scores in SAP populations, highlighting the need for developing disease-specific RFS risk prediction models.

Compared with previous studies, this is the first to focus on SAP patients—an extremely high-risk RFS population—and systematically compare multiple ML algorithms for model construction. For example, Zhan et al. (8) developed a well-discriminating model for general ICU patients, but included non-routinely monitored indicators (e.g., urinary epithelial cell count) with questionable applicability in SAP. Choi et al. (9) used XGBoost to predict refeeding hypophosphatemia, but the model targeted only one RFS component and included heterogeneous study populations, failing to reflect SAP-specific metabolic and inflammatory features. Zhang et al. (10) constructed an RFS prediction model for neurocritical patients, identifying risk factors such as drinking history and fasting duration, but the pathophysiological core (consciousness disorder-related nutritional intake impairment) differs fundamentally from SAP's pancreatitis-mediated digestive failure and hypercatabolism. Additionally, their model used traditional logistic regression, failing to capture complex non-linear relationships or quantify feature contributions via interpretability algorithms, limiting precision and acceptance in clinical translation. Our study fills the gap of SAP-specific RFS prediction models, with LASSO-selected predictors (e.g., electrolytes, gastrointestinal decompression) closely aligned with SAP clinical course, ensuring biological rationality and clinical relevance. Furthermore, SHAP analysis enhances the interpretability of the complex ML model, translating predictions into intuitively understandable clinical decision support. All predictors are derived from routine clinical data without requiring additional tests, ensuring excellent clinical practicality and wide applicability.

Nevertheless, this study has several limitations. First, the single-center, retrospective design and limited sample size may introduce selection bias. Center-specific practices such as enteral nutrition protocols and electrolyte monitoring strategies may affect model performance and generalizability. Future plans include multicenter external validation and single-center prospective validation, enrolling SAP patient cohorts from centers in different regions and with varying medical levels. The Bootstrap method will be used to calculate optimism-adjusted performance metrics for more rigorous evaluation of the model's generalization ability and clinical applicability. Second, this study focuses on SAP patients, so the model's extrapolation to mild and moderately severe acute pancreatitis patients requires caution; the SAP cohort was not further stratified by severity indicators such as Marshall score and duration of organ failure, and residual disease heterogeneity may impact model performance. Future studies will incorporate these severity indicators to optimize the model and expand its application scope through multicenter research. Third, due to the retrospective design, clinical data were not fully retrievable. Specific types and indications of diuretics were not collected, nor were details of nutritional support such as infusion rate and caloric targets—key modifiable factors in RFS management (26). Fourth, to improve the specificity of RFS diagnosis, this study adopted a stricter definition than the 2020 ASPEN criteria. While this helps reduce the inclusion of non-specific cases, it may exclude some early or mild RFS cases, potentially affecting the model's ability to identify the full spectrum of RFS risks. Future research can validate the model's performance under a broader definition.

## 5 Conclusion

In summary, this study developed and validated an explainable ML prediction model for RFS in SAP patients. The GBM model demonstrates satisfactory predictive performance and generalization ability, requiring only seven easily accessible clinical features. This tool enables early identification of high-risk SAP patients before nutritional support initiation, facilitating timely interventions and optimized nutrition management to improve patient outcomes.

## Data Availability

The original contributions presented in the study are included in the article/Supplementary material, further inquiries can be directed to the corresponding authors.
