# Supplementary material for: Explainable machine learning model to predict refeeding syndrome in patients with severe acute pancreatitis
Source: Front Nutr. 2026 Jan 23;12:1741052. doi: 10.3389/fnut.2025.1741052 (PMC12879345; doi:10.3389/fnut.2025.1741052)
Supplement: Supplementary file 1 [file Table_1.docx]

**Table S1.** Baseline characteristics of SAP patients across the two datasets

| **Variables** | **Total**  **(n = 461)** | **Training Set**  **(n=322)** | **Testing Set**  **(n=139)** | ***P* value** |
| --- | --- | --- | --- | --- |
| **RFS, n (%)** | 166 (36.01) | 121 (37.58) | 45 (32.37) | 0.285 |
| **Demographic** |  |  |  |  |
| Age, median[IQR] | 42.00 [33.00, 56.00] | 41.00 [33.00, 56.00] | 43.00 [34.00, 58.00] | 0.220 |
| Gender, Female, n (%) | 172 (37.31) | 115 (35.71) | 57 (41.01) | 0.281 |
| BMI, median [IQR] | 26.78 [24.22, 29.80] | 26.67 [24.01, 29.74] | 27.34 [24.50, 30.48] | 0.105 |
| Smoking history, n (%) | 102 (22.13) | 77 (23.91) | 25 (17.99) | 0.159 |
| Alcohol consumption, n (%) | 108 (23.43) | 79 (24.53) | 29 (20.86) | 0.393 |
| NRS, median[IQR] | 0.00 [0.00, 0.00] | 0.00 [0.00, 0.00] | 0.00 [0.00, 0.00] | 0.543 |
| **Comorbidities, n (%)** |  |  |  |  |
| Hypertension | 151 (32.75) | 100 (31.06) | 51 (36.69) | 0.237 |
| Diabetes | 163 (35.36) | 114 (35.40) | 49 (35.25) | 0.975 |
| LD | 8 (1.74) | 5 (1.55) | 3 (2.16) | 0.946 |
| CKD | 23 (4.99) | 13 (4.04) | 10 (7.19) | 0.153 |
| **Treatments, n (%)** |  |  |  |  |
| MV | 59 (12.80) | 36 (11.18) | 23 (16.55) | 0.113 |
| RRT | 172 (37.31) | 112 (34.78) | 60 (43.17) | 0.088 |
| Decompression | 132 (28.63) | 89 (27.64) | 43 (30.94) | 0.473 |
| Insulin | 285 (61.82) | 199 (61.80) | 87 (62.59) | 0.873 |
| Diuretics | 119 (25.81) | 78 (24.22) | 41 (29.50) | 0.235 |
| Glucocorticoids | 86 (18.66) | 53 (16.46) | 33 (23.74) | 0.066 |
| **Type, n (%)** |  |  |  | 0.117 |
| Biliary | 210 (45.55) | 147 (45.65) | 63 (45.32) |  |
| Alcoholic | 23 (4.99) | 21 (6.52) | 2 (1.44) |  |
| Hyperlipidemic | 90 (19.52) | 59 (18.32) | 31 (22.30) |  |
| Other | 138 (29.93) | 95 (29.50) | 43 (30.94) |  |
| **Laboratory results, median [IQR]** |  |  |  |  |
| TBIL, µmol/L | 14.00 [8.40, 23.40] | 14.05 [8.60, 23.17] | 13.60 [8.35, 24.05] | 0.957 |
| ALP, U/L | 82.00 [62.00, 108.00] | 81.50 [62.00, 110.50] | 84.00 [62.50, 106.00] | 0.934 |
| PALB, g/L | 0.11 [0.08, 0.14] | 0.11 [0.08, 0.15] | 0.10 [0.07, 0.13] | **0.018** |
| ALB, g/L | 32.70 [30.10, 35.40] | 32.80 [30.00, 35.20] | 32.50 [30.40, 35.50] | 0.804 |
| BUN, mmol/L | 5.20 [3.49, 8.26] | 5.19 [3.54, 7.82] | 5.26 [3.37, 9.84] | 0.462 |
| Scr, µmol/L | 53.00 [41.00, 70.00] | 53.00 [42.00, 68.00] | 51.00 [39.50, 79.50] | 0.974 |
| Chol, mmol/L | 4.58 [3.40, 6.38] | 4.68 [3.55, 6.54] | 4.34 [3.00, 6.04] | 0.078 |
| TG, mmol/L | 3.08 [1.55, 4.87] | 3.06 [1.74, 4.93] | 3.15 [1.43, 4.76] | 0.772 |
| K, mmol/L | 3.79 [3.45, 4.13] | 3.79 [3.45, 4.19] | 3.73 [3.46, 4.08] | 0.528 |
| Na, mmol/L | 138.10 [135.30, 142.50] | 138.00 [135.30, 142.00] | 138.50 [135.55, 144.00] | 0.323 |
| Mg, mmol/L | 0.83 [0.74, 0.92] | 0.83 [0.75, 0.92] | 0.83 [0.74, 0.92] | 0.746 |
| Ca, mmol/L | 2.01 [1.73, 2.13] | 2.02 [1.75, 2.14] | 2.00 [1.54, 2.13] | 0.483 |
| P, mmol/L | 0.78 [0.53, 1.01] | 0.78 [0.54, 1.01] | 0.79 [0.53, 1.02] | 0.689 |

Abbreviations: SAP, Severe Acute Pancreatitis; RFS, Refeeding Syndrome; IQR, Interquartile Range; BMI, Body Mass Index; NRS, Nutritional Risk Screening; LD, Liver Disease; CKD, Chronic Kidney Disease; MV, Mechanical Ventilation; RRT, Renal Replacement Therapy; TBIL, Total Bilirubin; ALP, Alkaline Phosphatase; PALB, Prealbumin; ALB, Albumin; BUN, Blood Urea Nitrogen; Scr, Serum Creatinine; Chol, Total Cholesterol; TG, Triglycerides; K, Potassium; Na, Sodium; Mg, Magnesium; Ca, Calcium; P, Phosphate.

**Table S2.** Hosmer-Lemeshow Test: *P*-values of each model in the training set and testing set

| **Dataset** | **LR** | **SVM** | **RF** | **GBM** | **XGBoost** | **EN** |
| --- | --- | --- | --- | --- | --- | --- |
| **Training set** | 0.235 | 0.202 | < 0.001 | 0.003 | 0.019 | 0.239 |
| **Testing set** | 0.245 | 0.356 | 0.154 | 0.202 | 0.091 | 0.270 |

Abbreviations: LR, Logistic Regression; SVM, Support Vector Machine; RF, Random Forest; GBM, Gradient Boosting Machine; XGBoost, eXtreme Gradient Boosting; EN, Elastic Network.
